# Recurrent exercise-induced acute renal failure in a young Pakistani man with severe renal hypouricemia and *SLC2A9* compound heterozygosity

**DOI:** 10.1186/1471-2350-15-3

**Published:** 2014-01-07

**Authors:** Guido Jeannin, Nicola Chiarelli, Mario Gaggiotti, Marco Ritelli, Paolo Maiorca, Stefano Quinzani, Federica Verzeletti, Stefano Possenti, Marina Colombi, Giovanni Cancarini

**Affiliations:** 1 Operative Unit of Nephrology, Department of Medical and Surgical Specialties, Radiological Sciences and Public Health, University Hospital Spedali Civili, Brescia, Italy; 2 Division of Biology and Genetics, Department of Molecular and Translational Medicine, University of Brescia, Viale Europa 11, 25123 Brescia, Italy

**Keywords:** Renal hypouricemia, Exercise-induced acute renal failure, *SLC2A9* mutations, p.Arg380Trp, p.Gly216Arg

## Abstract

**Background:**

Familial renal hypouricemia (RHUC) is a hereditary disease characterized by hypouricemia, high renal fractional excretion of uric acid (FE-UA) and can be complicated by acute kidney failure and nephrolithiasis. Loss-of-function mutations in the *SLC22A12* gene cause renal hypouricemia type 1 (RHUC1), whereas renal hypouricemia type 2 (RHUC2) is caused by mutations in the *SLC2A9* gene.

**Case presentation:**

We describe a 24-year-old Pakistani man who was admitted twice to our hospital for severe exercise-induced acute renal failure (EIARF), abdominal pain and fever; he had very low serum UA levels (0.2 mg/dl the first time and 0.09 mg/dl the second time) and high FE-UA (200% and 732% respectively), suggestive of RHUC. Mutational analyses of both urate transporters revealed a new compound heterozygosity for two distinct missense mutations in the *SLC2A9* gene: p.Arg380Trp, already identified in heterozygosity, and p.Gly216Arg, previously found in homozygosity or compound heterozygosity in some RHUC2 patients. Compared with previously reported patients harbouring these mutations, our proband showed the highest FE-UA levels, suggesting that the combination of p.Arg380Trp and p.Gly216Arg mutations most severely affects the renal handling of UA.

**Conclusions:**

The clinical and molecular findings from this patient and a review of the literature provide new insights into the genotype-phenotype correlation of this disorder, supporting the evidence of an autosomal recessive inheritance pattern for RHUC2. Further investigations into the functional properties of GLUT9, URAT1 and other urate transporters are required to assess their potential research and clinical implications.

## Background

Renal hypouricemia is a heterogeneous genetic disorder characterized by impaired tubular transport, reabsorption insufficiency and/or accelerated secretion of uric acid (UA) accompanied by severe complications, such as exercise-induced acute renal failure (EIARF), chronic kidney disease (CKD) and nephrolithiasis. Diagnosis of renal hypouricemia is based on biochemical markers, i.e., hypouricemia and increased fractional excretion of UA (FE-UA).

Renal hypouricemia type 1 (RHUC1, MIM#220150) is a recessive condition caused by mutations in the *SLC22A12* gene encoding the transporter URAT1, which leads to a partial UA absorption defect [1-5]. Renal hypouricemia type 2 (RHUC2, MIM#612076) is a disorder caused by defects in the *SLC2A9* gene encoding the facilitative glucose transporter 9 (GLUT9). Heterozygous RHUC2 patients show UA values similar to those of patients with URAT1 mutations, while homozygous and/or compound heterozygous patients have considerably lower serum UA levels (near 0 mg/dl) [normal range 2.35–7.90 mg/dl] and higher renal UA excretion (>100%) [normal range 4–14%] [6,7].

More than 100 patients with mutations in the *SLC22A12* gene have been described, whereas only a few patients with defects in the *SLC2A9* gene have been characterized. *SLC22A12* and *SLC2A9* are the two genes that most commonly influence the serum level of UA, exerting their effect by modifying renal UA absorption [1,8]. The *SLC22A12* gene encodes two transcript variants of the URAT1 transporter, and both variants are specifically expressed on the apical membrane of the proximal tubules in the kidneys [1].

The human *SLC2A9* gene encodes two isoforms of GLUT9, long and short, through the use of alternative promoters. GLUT9 is expressed in human kidney proximal tubule epithelial cells, in particular, GLUT9L might be localized to the basolateral side and GLUT9S to the apical membrane, as suggested by expression studies performed using the Madin-Darby canine kidney cells [9].

Dinour and coworkers speculated that UA efflux is mediated solely by GLUT9L on the basolateral side, whereas UA absorption from the tubular lumen is carried out not only by URAT1, but also by GLUT9S and possibly other apical transporters [10]. According to this hypothesis, in patients with RHUC1, loss-of-function of URAT1 should produce a partial UA absorption defect with a FE-UA of 40 to 90%, whereas loss-of-function of GLUT9, in RHUC2 patients, due to either homozygous or compound heterozygous mutations, should preclude UA absorption by all the apical transporters (including URAT1), through complete blocking of UA efflux, resulting in a total UA reabsorption defect with a FE-UA of >100% [10-12].

In patients with RHUC2, either heterozygous mutations [11,12] or compound heterozygous and/or homozygous mutations have been identified in *SLC2A9*[6,7,10,13,14].

To date, in *SLC2A9*, a total of 12 mutations (9 missense/nonsense, 1 small insertion, 1 gross insertion, and 1 gross deletion) are currently reported in the HGMD professional 2012.4.

Heterozygous *SLC2A9* mutations cause hypouricemia mainly via haploinsufficiency [11,12,15]. In a number of studies, homozygous and compound heterozygous loss-of-function mutations have been found to cause severe hypouricemia (serum UA near 0), markedly higher renal excretion, and nephrolithiasis and EIARF in most patients described from different ethnic groups [6,7,10,13,14]. Very recently, it was demonstrated that homozygous and/or compound heterozygous mutations do not necessarily lead to severe hypouricemia and a FE-UA of >150% [7].

Here, we report a young Pakistani man with severe EIARF who required temporary hemodialysis and who presented with very low serum UA levels. The FE-UA was high (>150%), confirming the clinical diagnosis of RHUC. The severe clinical manifestation of low UA and very high FE-UA were suggestive of RHUC2 secondary to compound heterozygous or homozygous mutations in *SLC2A9*. Mutational analysis of the *SLC22A12* and *SLC2A9* genes revealed the presence of two previously described mutations in the GLUT9 transporter, one found only in heterozygosis and the other either in compound heterozygosis or homozygosis, allowing RHUC2 diagnosis and providing new insights into the genotype-phenotype correlation in this disorder.

## Case presentation

A 24-year-old Pakistani man presented to the emergency room of our hospital suffering from diffuse abdominal and bilateral loin pain, diarrhea, vomiting and fever which began three days after a cricket match. The laboratory tests revealed kidney failure (serum creatinine 5.8 mg/dl; reference range 0.5-1.2), and the patient was admitted to our department. The patient appeared alert without neurological deficits, his temperature was 38°C, blood pressure was 146/75 mmHg, pulse was 72 beats per minute rhytmic and regular, respiratory rate was 16 breaths per minute. Complete laboratory tests confirmed acute renal failure (serum creatinine 6.3 mg/dl) with metabolic acidosis, hypokalemia and hyperphosphatemia. Urinalysis showed many UA crystals, microscopic hematuria and significant eosinophiluria (2%) without proteinuria. The patient’s serum UA was 2.9 mg/dl, creatine kinase was 1,155 U/l, and Prothrombin Time (PT) was 52% with an International Normalized Ratio (INR) of 1.6. All other laboratory tests were within the normal range (Table 1). Renal ultrasound showed only mild cortical hyperechogenicity, and renal vasculature was normal. The patient was not taking medications, and his medical history was unremarkable except for recurrent episodes of loin pain (frequently radiating to the right groin with fever and vomiting) over the last five years. These episodes had started frequently after cricket matches or other physical activities. The family history was unremarkable except for a 28-year-old sister, who was noted to have similar episodes of recurrent loin pain and vomiting.

**Table 1:** Patient’s laboratory tests at admission and discharge for both hospitalization episodes

**Serum parameters**	**Units**	**Reference values**	**1st admission**	**1st discharge**	**2nd admission**	**2nd discharge**
Hemoglobin	g/dl	14-18	13.4	13.8	14.7	14.2
Platelets	x10^3^/mmc	130-400	204	236	244	254
White blood cells	x10^3^/mmc	4-10.8	8.220	12.250	9.900	7.920
Eosinophils	%	0-8	3	0	2	
Aspartate transaminase	U/l	5-50	29	16	37	7
Alanine transaminase	U/l	5-50	13	11	16	12
Lactate dehydrogenase	U/l	125-220	191	208	233	115
Creatine kinase	U/l	20-170	1155	203	723	25
Serum proteins	g/dl	6-8	6.6		7	
C reactive protein	mg/l	< 5	15.4		17.5	
INR*		0.9-1.2	1.6	1.4	1.3	1.3
Partial thromboplastin time	seconds	24-38	38	40	34	35
Serum creatinine	mg/dl	0.5-1.2	12.0	1.6	2.1	0.9
Serum UA	mg/dl	2.35–7.90	2.9	0.2	0.38	0.09
Urine UA	mg/day	250-750		575		411
FE-UA	%			>150%		>150%
-men		6-12		(200%)		(732%)
-women		6-20				
Serum sodium	mmol/l	135-145	140	141	142	142
Serum potassium	mmol/l	3.5-5	3.3	3.5	3.5	3.6
Serum calcium	mg/dl	8.6-10.6	9.2	8.8	10.1	9.3
Serum phosphate	mg/dl	2.7-4.5	6.2	3.6	2.2	3.6
Venous HCO_3_^–^	mmol/l	24-28	19	26	26	30

*INR: International Normalized Ratio.

Intravenous infusions of 5% dextrose, normal saline and 1/6 M sodium bicarbonate were started. The next day, kidney function worsened further, and one hemodialysis session was performed.

A presumptive diagnosis of acute renal failure due to tubulo-interstitial nephritis was made, and prednisone therapy was started (67.5 mg/day, i.e., 1 mg/kg BW/day).

Kidney function gradually improved after the first dialysis session, and over the following nine days, there was complete recovery of renal function along with the disappearance of loin and groin pain and fever. Laboratory tests repeated before discharge were unremarkable, except for a very low plasma UA level (0.2 mg/dl). Steroid treatment was rapidly tapered off, and the patient was discharged on the thirteenth day with a serum creatinine level of 1.6 mg/dl.

Five months later, the patient presented again with recurrent loin pain, vomiting, diarrhea and acute renal failure (creatinine 2 mg/dl); two days before he had played a cricket match and the day before he had taken 1 g of paracetamol to treat a fever above 38°C.

The physical examination only showed bilateral loin tenderness; there were no signs of fluid overload, blood pressure was 121/68 mm Hg without orthostatic hypotension, and body temperature was 37°C.

The chest radiogram was normal, and renal ultrasonography showed normal-sized kidneys with only slightly increased echogenicity. Blood tests (Table 1) showed serum creatinine of 2.1 mg/dl, hypouricemia (0.4 mg/dl), hypophosphatemia (2.2 mg/dl) and a slight increase in creatine kinase (723 U/l). Urinalysis showed only rare red and white blood cells and a mild mixed proteinuria (0.4 g/day). Blood tests repeated after renal function recovery showed a very low serum UA concentration (0.09 mg/dl). The daily urine UA excretion was 411 mg with a FE-UA higher than 150%. This severe clinical manifestation, low UA and very high FE-UA in both admissions were suggestive of RHUC, which led us to perform mutational analyses of the two known casual genes. The patient provided written, informed consent and authorized the processing of his personal data according to Italian bioethics laws as well as the collection of blood and urine samples for biochemical and genetic analyses.

Genomic DNA was isolated from peripheral blood by standard protocol and all the exons and intron flanking regions of the *SLC22A12* gene (NM_144585.2, NP_653186.2) and of both *SLC2A9* isoforms (GLUT9L, NM_020041.2, NP_064425.2; GLUT9S, NM_001001290.1, NP_001001290.1) were analyzed by direct sequencing with the ABI3130XL Genetic Analyzer.

Sequence descriptions of the mutations were verified using the Alamut software, version 2.2.

**Figure 1:**
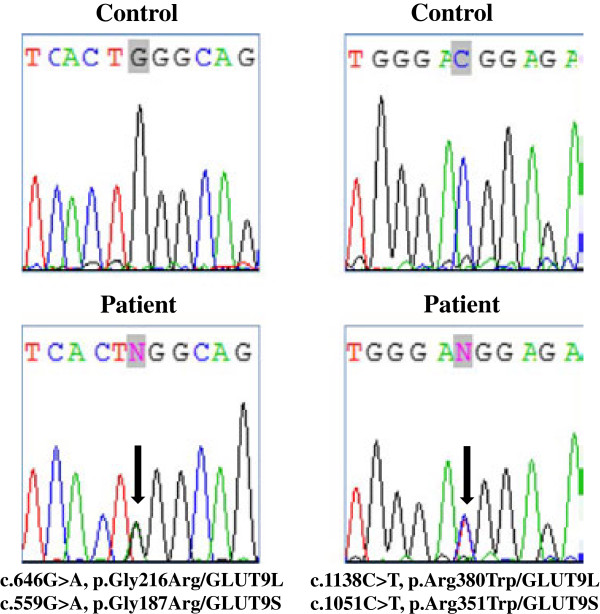
**Molecular characterization of the patient.** Left panel: Sequence chromatogram showing the position of the heterozygous c.646G>A transition (NM_020041.2, GLUT9L, exon 7) corresponding to c.559G>A (NM_00100290.1, GLUT9S, exon 7), leading to the p.Gly216Arg missense mutation (NP_064425.2, GLUT9L) corresponding to p.Gly187Arg (NP_001001290.1, GLUT9S). Right panel: Sequence chromatogram with the position of the heterozygous c.1138C>T transition (NM_020041.2, GLUT9L, exon 11) corresponding to c.1051C>T (NM_00100290.1, GLUT9S, exon 11), leading to the p.Arg380Trp missense mutation (NP_064425.2, GLUT9L, corresponding to p.Arg351Trp (NP_001001290.1, GLUT9S). Mutations are annotated according to HGVS nomenclature (http://www.hgvs.org/mutnomen). Nucleotide numbering is based on cDNA sequence numbering, with +1 corresponding to the A of the ATG translation initiation codon 1 in the reference sequence. For protein numbering, +1 corresponds to the first translated amino acid.

Sequencing analysis of *SLC22A12* did not identify casual mutations; however, in *SLC2A9,* two distinct missense mutations were identified (Figure 1). In particular, heterozygosity for p.Gly216Arg and p.Arg380Trp substitutions was identified. Both missense mutations have already been described in patients with RHUC2: the p.Gly216Arg mutation was found either in homozygosis or in compound heterozygosity [7], and the p.Arg380Trp substitution was found only in heterozygosity [11].

The phase of the p.Gly216Arg and p.Arg380Trp mutations was not determined given the long distance between the variants, and segregation analysis was not performed owing to the unavailability of the proband’s family.

The p.Arg380Trp mutation occurs in the sugar transport protein signatures 1/2, in the fourth cytoplasmic loop, which is highly conserved in all known GLUT9 orthologs [11]. Previous studies have shown that this arginine residue plays a critical role in forming cytoplasmic anchor points that are essential for the appropriate conformation of GLUT proteins [16]. Loss of cytoplasmic anchor points of the membrane topology results in a significant reduction in UA transport activity in mutated GLUT9. In addition, the p.Arg380Trp mutation was functionally characterized in the *Xenopus* model, where it demonstrated a marked reduction of UA transport activity compared to wild type GLUT9 [11,15].

The p.Gly216Arg mutation occurs in the fifth transmembrane domain and replaces a highly conserved glycine residue with a basic charged amino acid, which strongly suggests its pathogenicity, as also predicted by the computational tools of the Alamut software.

## Conclusions

We have described a 24-year-old Pakistani man with severe RHUC2 and EIARF. This clinical report emphasizes the importance of analyzing any unexpected data. Indeed, during the patient’s first admission, his low serum UA levels, as well as the presence of UA crystals in his urine, were underestimated, and did not immediately address the genetic testing for primary renal hypouricemia.

The differential diagnosis of EIARF usually includes massive rhabdomyolysis or severe hypovolemia. More rarely, some metabolic factor (hypo/hyperthyroidism, poorly controlled diabetes, hypocortisolism, and hypokalemia) can favor either hypovolemia or rhabdomyolysis. The environmental situation (altitude, humidity, and temperature), exercise intensity, medications or toxins (snake venom, insects, alcohol, stimulants, diet/herbal supplements, anticholinergic agents, statins, etc.) and infections can also cause rhabdomyolysis. In addition, genetic diseases should be considered (glycogen storage disorders, fatty acid oxidation disorders, mitochondrial disorders, sickle-cell anemia, and muscular dystrophy).

Our patient displayed neither significant rhabdomyolysis nor identifiable causes. His serum UA concentration was unexpectedly low during acute renal failure and decreased to very low values when renal function recovered.

Hypouricemia is arbitrarily defined as a serum UA concentration lower than 2 mg/dl (119 μmol/l). It is due to either decreased production (isolated defect in purine metabolism) or, more commonly, reduced renal tubular reabsorption of UA. The latter can be inherited (through mutation of genes encoding tubular reabsorption transporters), acquired as a result of disease (severe liver disease, malignancy, diabetes, acquired immunodeficiency syndrome, and syndrome of inappropriate antidiuretic hormone secretion) or as a result of drugs that enhance UA excretion.

Differential diagnosis between the different causes of hypouricemia is based on FE-UA. Hypouricemia with a high FE-UA is found in the familiar form of renal hypouricemia, diseases associated with Fanconi syndrome, hypervolemic states and diseases that increase intracranial pressure (cerebral salt-wasting syndrome) [17].

In our patient, a familial form of RHUC2 was considered based on the lack of other causes of hyperuricosuric hypouricemia, very low plasma UA, extremely elevated FE-UA (>150%), clinical presentation, and history of a sister with similar symptoms. EIARF associated with hyperuricosuric familial hypouricemia occurs predominantly in young males (male: female ratio of 8:1), mostly after strenuous exercise, such as a short-distance race. The initial symptoms are nausea, vomiting, loin pain, abdominal pain, general fatigue and low-grade fever.

Almost 25% of patients with RHUC2 experience recurrent EIARF at various intervals. Serum UA level is normal or only slightly reduced on presentation (due to renal failure) and decreases to less than 1 mg/dl after renal recovery, which usually occurs in all patients.

Our patient represents the most severe end of the spectrum of RHUC2 clinical and biochemical characteristics. In fact, *SLC2A9* analysis identified two distinct missense mutations (p.Gly216Arg and p.Arg380Trp); although segregation analysis was not performed, the severe clinical phenotype strongly suggests that they are present in compound heterozygosity. Both missense mutations have already been described in patients with RHUC2, but they have never been identified in combination in a single patient.

In previously documented patients, the recessive p.Gly216Arg mutation was either homozygous or in compound heterozygous patients [7], whereas the p.Arg380Trp substitution was observed only in heterozygosity and was therefore classified as a dominant mutation [11,15]. Comparison of the clinical and biochemical characteristics of our patient with the other recently reported patients provides new insights into the genotype-phenotype correlation of *SLC2A9* mutations associated with UA homeostasis. The p.Arg380Trp substitution was one of the first causal mutations identified in the *SLC2A9* gene, being found in heterozygosity in a Japanese mother and her son who had serum UA levels of 1.5 and 2.7 mg/dl respectively, and an FE-UA of ~15% [11]. These two patients were selected from the large database of the personnel of the Japan Maritime Self-Defense Force on the basis of UA levels, and severe clinical manifestations were not recorded. Therefore, this apparently dominant mutation alone is probably insufficient to cause a complete clinical phenotype of the disease, i.e., EIARF and hospitalization (Table 2).

**Table 2:** Molecular and clinical features of all RHUC2 patients reported to date

***SLC2A9 *** **mutations**	**Status**	**Gender**	**Age**	**Serum UA**	**FE-UA**	**Clinical manifestations**	**References**
**(GLUT9L, NP_064425.2)**			**(years)**	**(mg/dl)**	**(%)**		
p.Pro412Arg/WT	Heterozygous	Female	36	2.4	NA	No	[12]
p.Arg380Trp/WT	Heterozygous	Female	70	1.5	15.7%	No	[11]
		Male	43	2.7	14.6%	No	
p.Arg198Cys/WT	Heterozygous	Female	32	2.1	NA	No	[11]
p.Leu75Arg/p.Leu75Arg	Homozygous	Male	67	0.67	>150%	Nephrolithiasis, CKD	[10]
		Male	46	0.20	>150%	EIARF	
		Male	36	0.04	>150%	Nephrolithiasis	
		Female	10	0.01	>150%	No	
		Male	24	0.2	>150%	EIARF	
		Male	19	0.1	>150%	EIARF	
p.Leu75Arg/WT	Heterozygous	Female	64	4.5	5.4%	No	[10]
		Female	28	2.0	21.7%	No	
		Female	38	2.2	19.6%	No	
		Female	48	3.4	7.5%	No	
		Female	40	3.7	7.4%	No	
		Female	44	3.1	12.4%	No	
		Male	5	2.6	NA	No	
		Female	15	2.4	NA	No	
		Male	16	2.0	17%	No	
delExon7/delExon7	Homozygous	Male	69	0.1	>150%	Nephrolithiasis	[10]
p.Gly236*/dupExon1a-11	Compound heterozygous	Female	11	0.1	>150%	EIARF	[13]
p.Gly236*/WT	Heterozygous	Female	46	3.4	NA	No	[13]
dupExon1a-11/WT	Heterozygous	Male	52	4.8	NA	No	[13]
p.Ile119Hisfs*27/p.Ile119Hisfs*27	Homozygous	Female	16	0.17	>150%	No	[6]
		Male	21	0.17	>150%	No	
p.Ile119Hisfs*27/WT	Heterozygous	Male	NA	5.6	7.6%	No	[6]
		Female	NA	2.9	12.8%	No	
p.Arg171Cys/p.Arg171Cys	Homozygous	Female	7.5	0.1	138%	No	[14]
		Male	5.5	0.1	157%	No	
		Female	2.3	0.2	88.8%	No	
p.Arg171Cys/WT	Heterozygous	Female	24	3.8	3.2%	No	[14]
		Male	37	4.9	6.5%	No	
p.Thr125Met/p.Thr125Met	Homozygous	Male	84	0.2	>150%	No	[14]
p.Gly216Arg/p.Asn333Ser	Compound heterozygous	Male	14	0.67	93%	EIARF	[7]
p.Gly216Arg/p.Gly216Arg	Homozygous	Male	12	0.5	46%	EIARF	[7]
p.Gly216Arg/p.Arg380Trp	Compound heterozygous	Male	24	0.1	>150%	EIARF	This study

NA: not available; WT: wild type.

The p.Gly216Arg substitution was originally described in a 14-year-old boy in compound heterozygosity with the p.Asn333Ser mutation [7]. The patient presented with EIARF (maximum serum creatinine level of 297 μmol/l) following a cross-country run, and his serum UA concentration was consistently below the normal range at 0.67 mg/dl (normal range 2.35–7.90 mg/dl), daily urinary UA excretion was 2190 mg (normal range 250–750 mg), and FE-UA was 93% (normal range 4–14%). The renal recovery was complete, and after the original episode, he had further incidences of mild abdominal pain and mild renal dysfunction following exercise. The p.Gly216Arg mutation was also found in homozygosity in a 12-year-old patient presenting with severe EIARF requiring dialysis, abdominal pain and vomiting. His serum UA was found to be persistently low at 0.5 mg/dl, his urinary UA excretion was 2050 mg/die, and FE-UA was 45.8%; the patient recovered normal renal function. Both of these patients presented with severe EIARF, similar to our patient; however, their UA levels were higher (0.67 and 0.5 mg/dl *vs.* 0.1 mg/dl) and the FE-UA was significantly lower (93% and 45.8% *vs.* >150%) compared with our patient. Therefore, we can speculate that the p.Arg380Trp substitution most severely impairs the GLUT9 function in comparison with the p.Gly216Arg and p.Asn333Ser mutations, giving our compound heterozygous patient an increased risk of EIARF.

To date, several studies have described the clinical and molecular characteristics of different patients with a severe type of RHUC2 caused by compound heterozygous or homozygous loss-of-function mutations in the *SLC2A9* gene, suggesting an autosomal recessive inheritance pattern for RHUC2 (Table 2). In particular, Dinour et al. [10] and Shima et al. [13] reported patients that were either homozygous or compound heterozygous for mutations in *SLC2A9*, who showed severe hypouricemia (0.1-0.7 mg/dl), very high FE-UA (>150%) and a high incidence of EIARF, nephrolithiasis and CKD compared with the asymptomatic heterozygous carriers, who showed only moderately low serum UA levels (Table 2).

The data presented here confirm that loss-of-function mutations in *SLC2A9* preclude UA absorption by all of the apical transporters (including URAT1). Additional factors, such as sequence variation in other UA transporters (*SLC22A12, SLC17A3, ABCC4* and *ABCG2*), age, sex, diet, drugs, physical activity, environment and volume status can influence the degree of UA tubular absorption and the risk of EIARF or nephrolithiasis. These factors could explain the 7/17 (41%) reported asymptomatic patients with homozygous/compound heterozygous *SLC2A9* mutations that show very low serum UA levels without clinical complications (Table 2) [6,10,14].

The pathogenesis of EIARF is unclear. There are two main hypotheses: one based on UA precipitation and the other on its antioxidant activity. The UA precipitation hypothesis proposes that if patients are exposed to a UA load, they are likely to have exaggerated excretion of UA. Prolonged or severe exercise is associated with an elevation in UA production and urinary excretion. If this condition is associated with hypovolemia, increased urine concentration and low urine pH, it favors the precipitation and crystallization of UA. The occurrence of UA nephrolithiasis in patients with renal hypouricemia and the finding of renal tubular obstruction by UA crystals in one patient with EIARF support a pathogenetic role for UA precipitation [18]. In our case this pathogenetic mechanism is supported by the findings of relative hypouricemia, urate crystals and red blood cells in urine and loin pain during the most severe episode of EIARF.

The second pathogenetic hypothesis is based on the powerful antioxidant activity of UA [6,7]. However, this hypothesis is in discrepancy with the evidence that ARF does not usually occur in patients with low hypouricemia due to classical xanthinuria, either type I, deficient in xanthine dehydrogenase (XDH) activity (MIM#278300); or type II, deficient in both XDH and aldehyde oxidase (MIM#603592) [19]. This suggests that hypouricemia alone, probably, could not contribute to ARF in patients with primary renal hypouricemia.

One could speculate why our patient had only two episodes of EIARF at the age of 24 and not all the times he had anaerobic physical activity. Possibly not all the exercises cause renal failure; other factors like duration and strength of the exercise, environmental temperature, relative humidity, state of normal or reduced hydration of the subject, changes in urine pH due to different foods and use of some drugs could play a role. Due to the severity of renal hypouricemia, our patient was instructed to restrict strenuous anaerobic exercise and take sufficient water after exercise. To prevent recurrence of EIARF prophylaxis with allopurinol, oral supplementation of antioxidants or scavengers, such as glutathione, vitamins C and E, and beta-carotene, are also recommended [20].

In conclusion, the clinical and molecular findings on the present patient provide new insights into this disorder, which is understood to a limited degree owing to the small number of patients with *SLC2A9* mutations described to date. Further investigations of the functional properties of GLUT9, URAT1 and other urate transporters are required to assess their potential research and clinical implications.

### Consent

Written informed consent was obtained from the patient for publication of this Case report and any accompanying images. A copy of the written consent is available for review by the Editor of this journal.

## Abbreviations

CKD: Chronic kidney disease; EIARF: Exercise-induced acute renal failure; FE-UA: Fractional excretion of uric acid; RHUC: Renal hypouricaemia; UA: Uric acid.

## Competing interests

The authors declared they have no competing of interests.

## Authors’ contributions

GJ, NC, MR, MC and GC designed the study. GJ, MG, PM, FV, SP and GC made the clinical diagnosis of the patient and performed the follow-up. MR, NC and SQ performed the molecular analyses. MR, NC, GJ, MC and GC researched the literature, reviewed and prepared the manuscript. MC and GC edited and coordinated the manuscript. All of the authors discussed, read, and approved the final manuscript.

## Pre-publication history

The pre-publication history for this paper can be accessed here:

http://www.biomedcentral.com/1471-2350/15/3/prepub
